# Acetylated Pyrimidine Metabolism Genes as Prognostic Markers and Their Influence on Immune Profiles in Lung Adenocarcinoma

**DOI:** 10.1155/humu/1500755

**Published:** 2026-01-21

**Authors:** Kegang Jia, Shuwei Zhang, Yangke He, Liang Liang

**Affiliations:** ^1^ Department of Thoracic Surgery, Sichuan Provincial People′s Hospital, University of Electronic Science and Technology of China, Chengdu, China, uestc.edu.cn; ^2^ Department of Oncology, Sichuan Provincial People′s Hospital, School of Medicine, University of Electronic Science and Technology of China, Chengdu, China, uestc.edu.cn

**Keywords:** acetylation, immunotherapy, lung adenocarcinoma, prognostic biomarkers, pyrimidine metabolism

## Abstract

Lung adenocarcinoma is a very aggressive cancer with poor clinical results. New molecular indicators are desperately needed to improve treatment decision‐making. This study looks at the relationship between the immunological microenvironment and genes linked to pyrimidine metabolism, particularly those that undergo acetylation, and the prognostic significance of these genes. Using publicly accessible genomic and clinical data, we used Gene Set Variation Analysis (GSVA) to identify acetylated pyrimidine pathway components that are highly correlated with survival outcomes. Three potential genes—TK1, RRM2B, and NME4—were identified for their prognostic relevance by using sophisticated predictive modeling approaches including CoxBoost and a random forest survival analysis. Using CIBERSORT deconvolution and single‐sample gene set enrichment, immunological landscape disparities were identified, and it was discovered that varied gene expression‐acetylation patterns were linked to varying immune cell infiltration. Gene activity and acetylation status‐based low‐risk patients showed positive survival patterns and higher levels of antitumor immune populations, indicating possible receptivity to immune‐based treatments. Functional validation experiments targeting TK1, including RNA interference followed by proliferation (CCK‐8, EdU), migration (Transwell), and wound healing assays, substantiated its role in promoting tumor aggressiveness. Collectively, our findings suggest that integrating metabolic gene signatures with immunological context offers a promising framework for precision oncology in lung adenocarcinoma.

## 1. Introduction

The most common subtypes of NSCLC lung adenocarcinoma contribute to high‐death rates globally and pose a serious threat to global health [1–3]. The prognosis for patients with lung adenocarcinoma is still poor, with a 5‐year survival rate of about 15%–20% across all stages of the illness, despite improvements in both diagnostic and treatment techniques [4, 5]. The disease′s propensity to be discovered at an advanced stage, its potential for metastasis, and the emergence of resistance to traditional treatments are the main causes of this dismal result [6, 7]. To increase the survival and quality of life for patients with this aggressive malignancy, there is a pressing need for better diagnostic instruments, prognostic indicators, and novel treatment targets [8].

By using genomic profiling to customize treatments for each patient, recent advancements in precision medicine have completely changed the way cancer is treated [8, 9]. Because of their potential to identify patients who may respond well to particular therapies, biomarkers have drawn a lot of interest within this paradigm [10]. As possible biomarkers for lung adenocarcinoma, genetic mutations and the expression levels of different genes have been thoroughly investigated [11–13]. Despite these developments, a sizable fraction of patients continue to have inferior clinical outcomes, highlighting the need for more research into tumor biology and the discovery of new biomarkers that might enhance treatment approaches.

The investigation of metabolic pathways that propel tumor growth and contribute to treatment resistance is one potential strategy for comprehending the pathophysiology of lung adenocarcinoma [14–16]. The metabolism of cancer cells frequently deviates greatly from that of healthy cells, with modified pathways facilitating fast cell division and survival, particularly in the presence of stressors such as hypoxia and nutritional shortage that are typical of the tumor microenvironment [17–20]. Because of its crucial function in DNA synthesis and repair, pyrimidine metabolism has garnered interest among the many metabolic changes and might be a target for innovative therapeutic approaches [20, 21]. The importance of pyrimidine metabolism as a therapeutic target has been highlighted by the identification of dysregulation of this metabolism in a number of malignancies, including lung adenocarcinoma.

Posttranslational modifications like acetylation have been identified as important factors in cancer biology in addition to metabolic changes [22]. The metabolic properties of cancer cells can be influenced by the acetylation of proteins, especially metabolic enzymes, which can significantly alter their stability and activity [22, 23]. According to recent research, alterations in the acetylation patterns of enzymes engaged in metabolic pathways may have a direct correlation with tumor behavior and patient outcomes, potentially providing new treatment approaches [23, 24].

Investigating the prognostic importance of acetylated genes implicated in pyrimidine metabolism in lung cancer is the goal of this investigation. We aim to find potential prognostic biomarkers and targetable pathways that may enhance the effectiveness of current treatments or guide the creation of novel therapeutic approaches by identifying acetylated genes within this metabolic pathway that are linked to patient survival.

It has also been demonstrated that the immunological milieu inside tumors is essential for the development of cancer and its reaction to treatment [24, 25]. Tumor growth and response to therapy are greatly impacted by the very varied makeup and behavior of tumor‐infiltrating immune cells in lung adenocarcinoma [25, 26]. Lung adenocarcinoma is among the numerous tumors for which immunotherapy, and immune checkpoint inhibitors in particular, have greatly improved treatment options; yet, its efficacy varies, with some patients exhibiting little to no response [26, 27]. It may be possible to gain important insights into the mechanisms behind these differences in treatment results by comprehending the connections between the tumor immunological milieu and metabolic processes, such as those controlled by genes involved in acetylated pyrimidine metabolism.

Finding important genes involved in acetylated pyrimidine metabolism linked to lung cancer survival outcomes and investigating their connection to the tumor immune milieu are the objectives of this study. Through the integration of gene expression data, immune infiltration analysis, and clinical outcomes, our proposal is to create a comprehensive model that forecasts the prognosis of patients and the responses to therapy, namely, immunotherapy. Through acetylation, our work will improve our knowledge of metabolic reprogramming in cancer, demonstrate its therapeutic potential, and offer fresh approaches to cancer treatment that target both metabolic and immunological pathways. Additionally, by utilizing cutting‐edge bioinformatics methods to examine extensive datasets, we want to close the gap between research findings in the lab and clinical settings, opening the door for tailored combination medicines in the treatment of cancer.

## 2. Materials and Methods

### 2.1. Study Design and Data Sources

This study examined the predictive significance of acetylated pyrimidine metabolism genes in lung cancer using a multistep computational and experimental methodology. To facilitate both discovery and external validation, publicly accessible transcriptomic and clinical datasets were acquired from GEO (https://www.ncbi.nlm.nih.gov/gds/?term=) and TCGA (https://portal.gdc.cancer.gov/). Utilizing the TCGA‐LUAD dataset, a differential gene expression analysis was conducted, which included 530 tumor samples and 59 nearby normal lung tissue samples. A total of 472 samples with complete survival data were chosen for the building of the prognostic model. GSE30219 (*n* = 85) and GSE42127 (*n* = 133) [27, 28], two separate validation cohorts with annotated survival data, were included. Two distinct gene sets were curated for this study. The first set included 84 genes associated with metabolic pathways derived from KEGG annotations. The second comprised 36 acetylation‐regulatory genes identified from literature describing acetylation‐driven malignancy patterns in hepatocellular carcinoma [29].

### 2.2. Pathway Activity Estimation

Using the GSVA and GSEA Base packages in R, Gene Set Variation Analysis was performed to assess functional pathway activity across samples. Pathway enrichment might be measured at the sample level using this nonparametric, unsupervised approach.

### 2.3. Clustering and Dimensionality Reduction

The Consensus Cluster Plus program was used to apply unsupervised consensus clustering to stratify patients according to pathway‐level activity profiles. Then, using the FactoMineR and factoextra programs, principal component analysis (PCA) was performed to investigate the variance and separability of the subgroups that were found.

### 2.4. Survival Analysis

To evaluate prognostic variations among patient subtypes, the survival and survminer packages employed log‐rank tests and Kaplan–Meier survival curves.

### 2.5. Heatmaps and Differential Expression

Heatmaps of metabolic activity were made using pheatmap to illustrate differences across subgroups. The clusterProfiler was utilized to conduct functional enrichment analysis on strongly deregulated genes, whereas the limma program was utilized to investigate differential gene expression. Both the Gene Ontology Biological Processes (GOBPs) and HALLMARK gene sets were evaluated. To aid with intuitive interpretation, visualization was done using the GseaVis program. The mutation annotation data were retrieved using the TCGA biolinks R program and shown using the maftools package. The mutational landscape was described using waterfall plots.

### 2.6. Univariate Cox Regression and Visualization

Using the survival software, single‐variable Cox regression was used to calculate the effect of each gene on survival. Correlation heatmaps were created using ggplot2, and Enhanced Volcano was used to create volcano visualizations. Bubble plots (ggrepel), split violin plots (ggpubr and ggsci), and comparison dot plots (ggpubr and rstatix) were used to visualize expression differences.

### 2.7. Prognostic Model Construction

Two machine learning‐based survival models were developed to identify key prognostic genes for lung adenocarcinoma: one using CoxBoost (via the CoxBoost package) and the other using a random forest survival analysis (via the randomForestSRC package). The gene sets identified by these models included four core prognostic genes, which were ranked according to their predictive power.

To generate the Acetylated Pyrimidine Gene (ARPG) score, the expression levels of three core genes (NME4, RRM2B, and TK1) were used. The score was calculated using a weighted linear combination of gene expression values, with the following formula:

ARPG Score=β142231×Expression of NME+β×Expression of RRMB+β×Expression of TK

(*β*1, *β*2, and *β*3 represent the coefficients derived from the CoxBoost model, reflecting the relative importance of each gene in predicting survival.)

Cross‐validation was performed within the TCGA‐LUAD dataset to ensure the model′s robustness and avoid overfitting. External validation was subsequently performed using independent cohorts, GSE30219 and GSE42127, where the ARPG score demonstrated a consistent predictive performance.

The overlap of gene candidates identified in both survival models was further explored using Venn diagrams, which revealed three common core genes: TK1, RRM2B, and NME4. The interactions between these genes were validated through Protein‐Protein Interaction (PPI) network analysis using the STRING database, confirming their potential role in the prognosis of lung adenocarcinoma.

### 2.8. Immune Profiling and Drug Response

CIBERSORTx was used to infer the makeup of immune cells using bulk RNA‐seq. Alluvial plots (ggalluvial) were used to illustrate longitudinal variations, whereas ggpubr was used to visualize immune infiltration levels. The integration of survival data and immunological markers was made easier by the IOBR software. pRRophetic was used to model genomic‐based drug response predictions, and boxplots and scatter plots were used to display the results.

### 2.9. Clinical Outcome Modeling

Associations between clinical parameters and survival were assessed via univariate Cox models and visualized with forest plots (ggplot2). Nomograms were constructed using the rms package to integrate risk scores and clinical factors. The timeROC package was used to evaluate model accuracy over time. Forest plots (ggplot2) were used to depict the relationships between clinical factors and survival, which were evaluated using univariate Cox models. The RMS software was used to create nomograms that included clinical criteria and risk scores. The accuracy of the model was assessed over time using the timeROC program.

### 2.10. In Vitro Validation Experiments

#### 2.10.1. Cell Culture and Transfection

We purchased A549 human lung adenocarcinoma cells from the Chinese Academy of Sciences′ Cell Bank. The cells were kept in RPMI‐1640 media (Thermo Fisher Scientific, United States) with 1% penicillin–streptomycin and 10% fetal bovine serum (FBS) added. They were then incubated at 37°C with 5% CO_2_. The TK1 gene was silenced using siRNA (Gima, China) in combination with Lipofectamine 3000 (Thermo Fisher, United States) after standard transfection protocols.

#### 2.10.2. RNA Isolation and RT‐qPCR

Total RNA from transfected cells was extracted using trizol (Takara, Japan).

Chloroform phase separation and isopropanol precipitation were then performed. Thermo Fisher Scientific′s Nano Drop 2000 was used to assess the purity of the RNA. For cDNA synthesis, the Prime Script RT kit (Takara, Japan) was utilized, and for RT‐qPCR, SYBR Green ER chemicals were employed. Relative expression levels were calculated using the 2^−*ΔΔ*Ct approach after adjusting TK1 expression to *β*‐actin. Each experiment had three biological replicates.

#### 2.10.3. Cell Proliferation Assays

The CCK8 kit (KeyGEN, China) was used to measure cell viability 24, 48, and 72 h after transfection. A microplate reader was used to measure absorbance at 450 nm. The EdU incorporation test (Beyotime, China) was used to quantify DNA synthesis, and fluorescence microscopy and DAPI labeling were the next steps.

#### 2.10.4. Migration and Invasion Assays

Cell invasion was evaluated using Matrigel‐coated transwell chambers. Noninvading cells were eliminated after a day, and migrating cells were examined under a microscope after being stained with 0.1% crystal violet. Using a wound‐healing experiment, migration potential was evaluated. To determine wound closure rates, pictures were obtained at 0 and 48 h after the scratch was created.

### 2.11. Ethical Compliance

Permission from the Institutional Review Board was not necessary because all of the data utilized in this study was taken from publicly accessible databases. Cell‐based investigations were conducted without the use of humans or animals.

### 2.12. Statistical Analysis

Statistical analyses were conducted using R (Version 4.0.0) and relevant packages. Differential gene expression was analyzed with limma, and survival differences were assessed using Kaplan–Meier curves and log‐rank tests (survival and survminer). Cross‐validation within the TCGA‐LUAD cohort was performed using CoxBoost and randomForestSRC. Performance was evaluated with time‐dependent ROC curves and AUC. Batch effects between TCGA and GEO datasets were mitigated using ComBat normalization from the sva package prior to model validation. A *p* value of < 0.05 was considered significant.

#### 2.12.1. GSVA‐Based Metabolic Profiling and Patient Stratification

To explore metabolic heterogeneity in lung adenocarcinoma, GSVA was conducted using 84 metabolism‐associated pathways across patient samples. The resultant enrichment scores were utilized for unsupervised consensus clustering. K = 2 produced optimal clustering, dividing the cohort into two discrete molecular subgroups (Figure 1a,b). An obvious divergence in these subgroups′ distributions was validated by PCA (Figure 1c). When compared with Cluster 1, patients in Cluster 2 had a noticeably longer overall survival, according to survival analysis (Figure 1d). According to a heatmap showing pathway activity, most metabolic pathways were elevated in Cluster 2 (Figure 1e), indicating that improved prognosis may be associated with increased metabolic performance.

Figure 1Metabolic pathway activity and patient stratification. (a): Hierarchical clustering heatmap of 84 metabolic pathways in LUAD patients, grouped into two clusters based on GSVA pathway scores. (b): Dendrogram from unsupervised hierarchical clustering showing patient division into two clusters based on metabolic pathway activity. (c): PCA plot illustrating the separation of the two clusters along the first two components, highlighting distinct metabolic profiles. (d): Kaplan–Meier survival curves comparing overall survival between Cluster 1 and Cluster 2. *p* values are derived from the log‐rank test. (e): Heatmap showing differential pathway activity between the two clusters, with pathways upregulated in Cluster 2 correlating with better survival.

**Figure:**
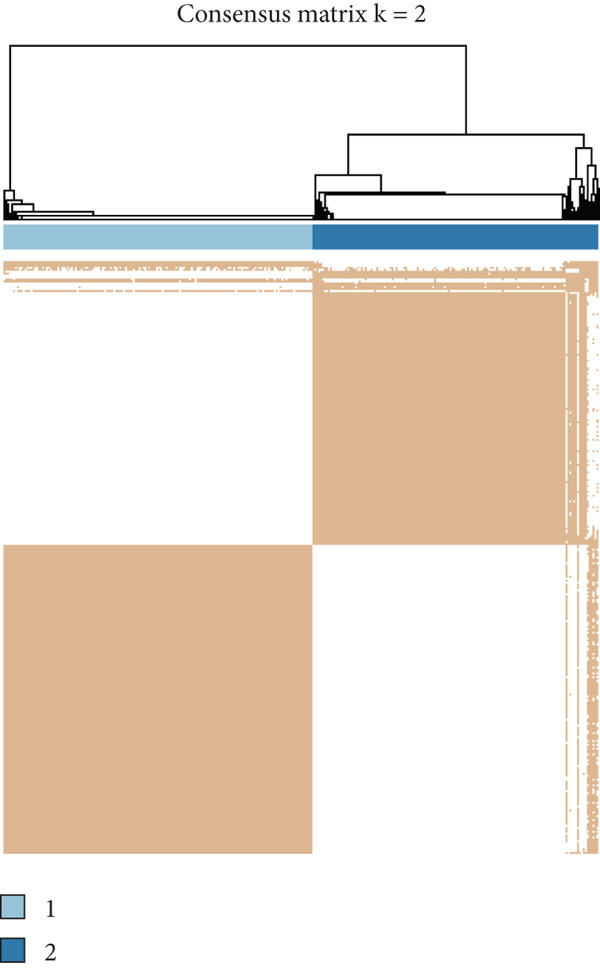
(a)

**Figure:**
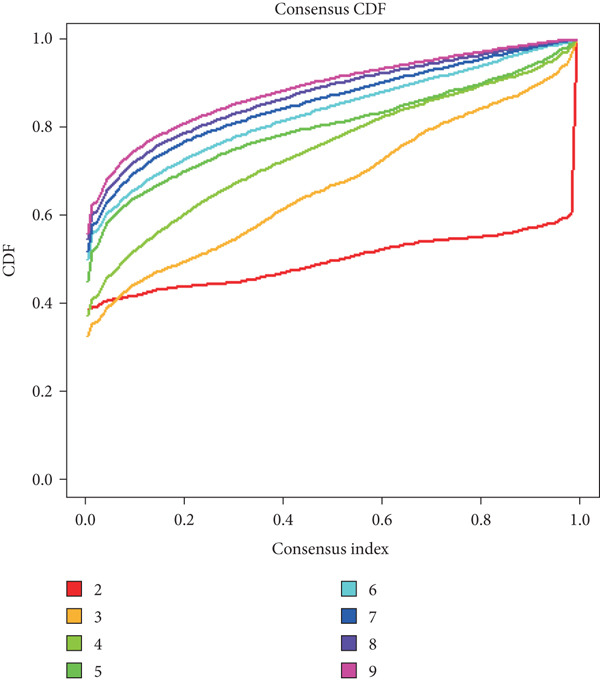
(b)

**Figure:**
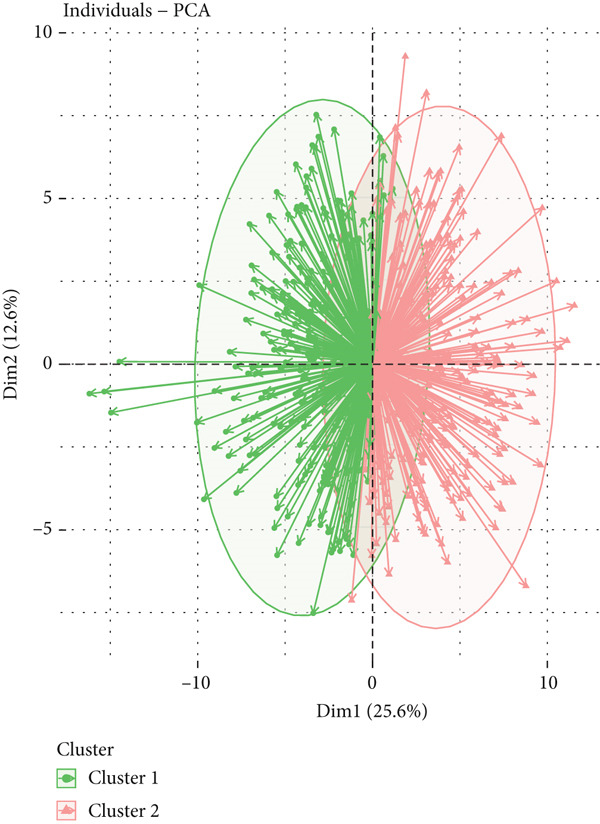
(c)

**Figure:**
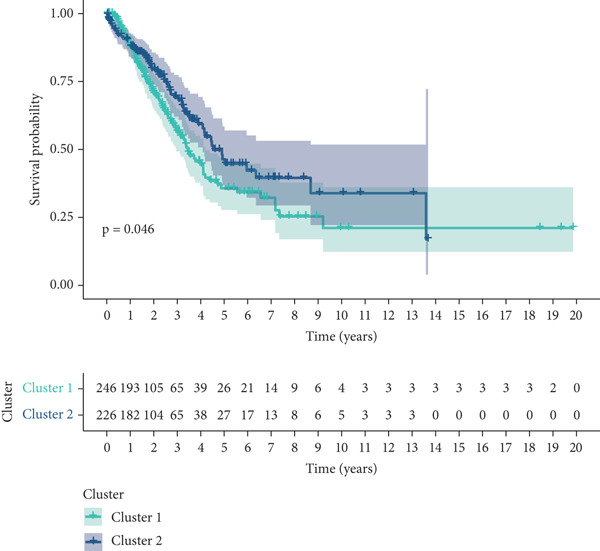
(d)

**Figure:**
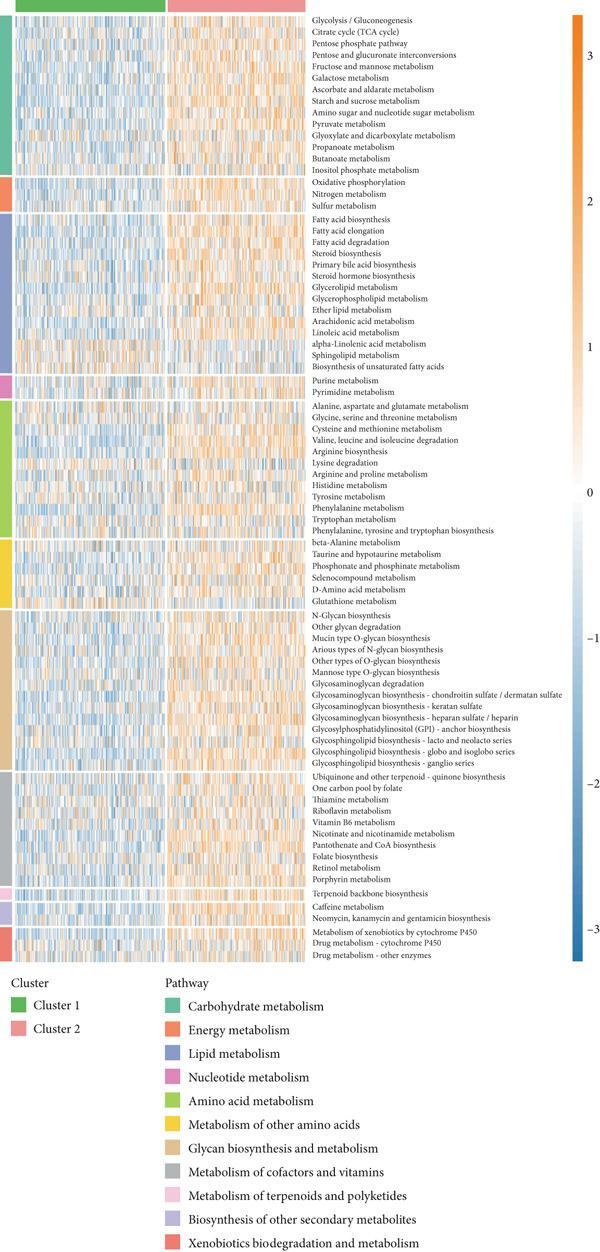
(e)

#### 2.12.2. Transcriptomic Differences and Functional Pathway Insights

Following an examination of the changes in gene expression between the two patient subtypes, enrichment analysis was carried out using the HALLMARK and GOBP databases. The GOBP analysis indicated enrichment in pathways such as lymphocyte activation regulation, oxidative phosphorylation, and fatty acid catabolism (Figure 2a). According to HALLMARK enrichment, Cluster 1 showed increased expression in E2F target genes and pathways linked to epithelial–mesenchymal transition, whereas Cluster 2 showed increased activity in oxidative phosphorylation, lipid biosynthesis, xenobiotic detoxification, and bile acid metabolism (Figure 2b,c). TP53 changes were detected in 58% of samples in Cluster 1 compared with 39% in Cluster 2, indicating a greater mutation burden in Cluster 1 according to somatic mutation profiling (Figure 2d,e).

Figure 2Gene set enrichment analysis and mutation profiles. (a): GSEA of GOBP showing pathways significantly enriched in each cluster, including oxidative phosphorylation and fatty acid metabolism. (b): Hallmark GSEA enrichment plot showing upregulated pathways in Cluster 2, including oxidative phosphorylation and bile acid metabolism. (c): Hallmark GSEA plot for Cluster 1 showing upregulated pathways like epithelial–mesenchymal transition and E2F targets. (d): Mutation profiles in the clusters, with higher TP53 mutation frequency in Cluster 1. (e): Bar graph depicting the percentage of mutations in top genes across clusters, with TP53 mutations more frequent in Cluster 1.

**Figure:**
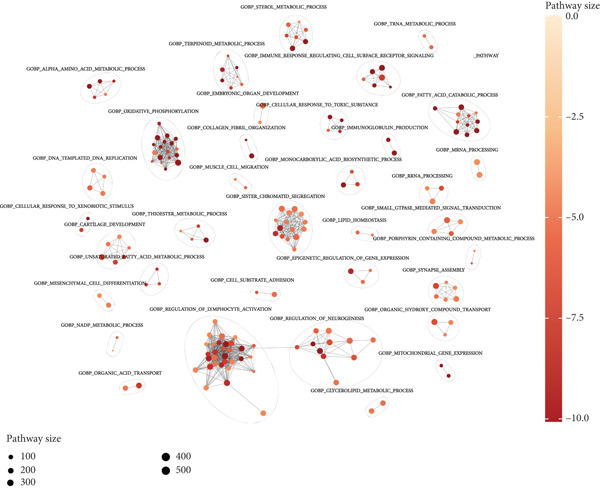
(a)

**Figure:**
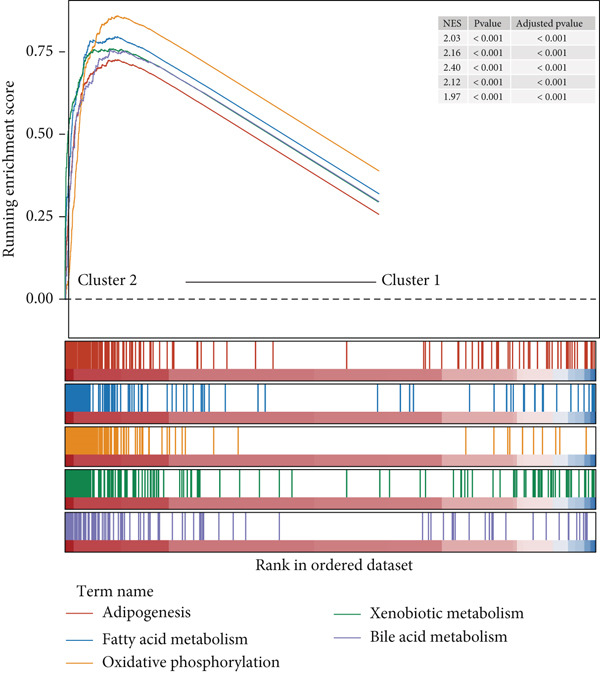
(b)

**Figure:**
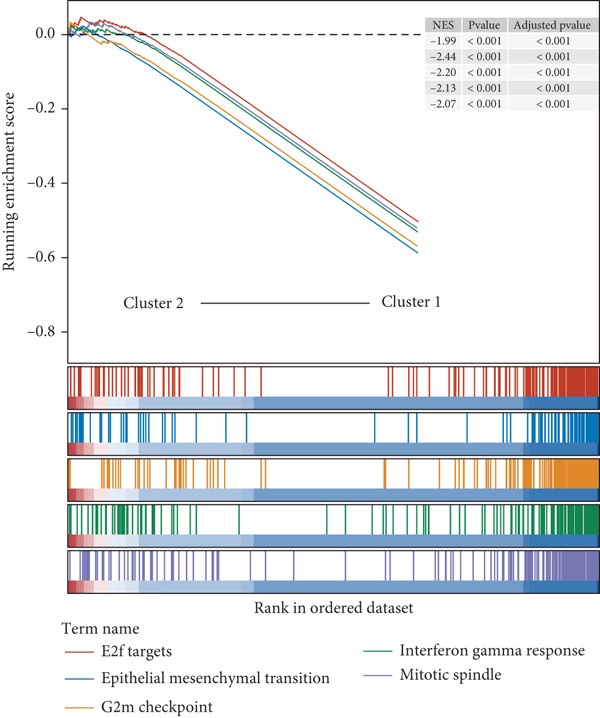
(c)

**Figure:**
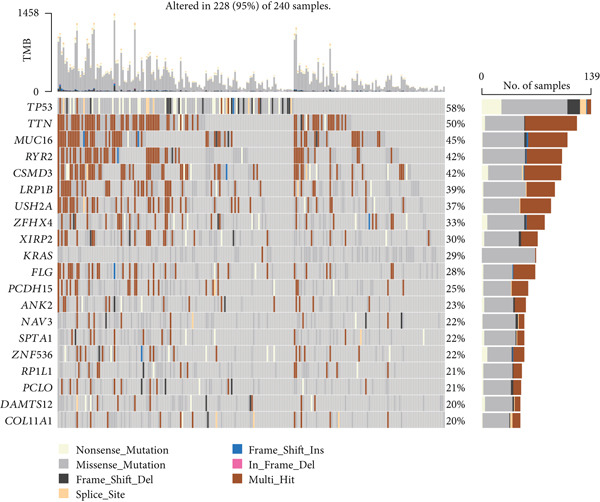
(d)

**Figure:**
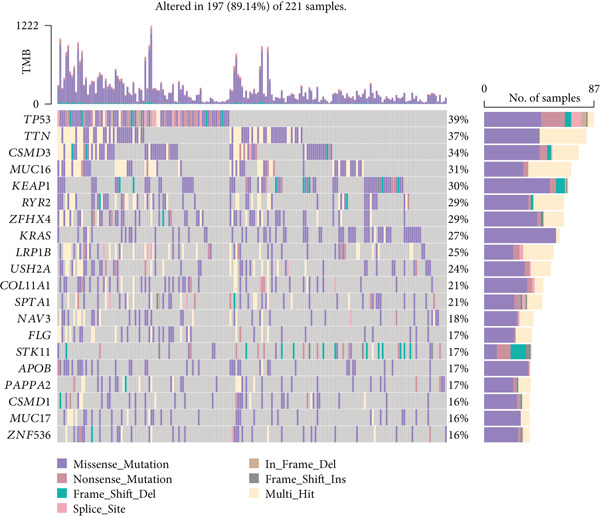
(e)

#### 2.12.3. Pyrimidine Metabolism Acetylation and Prognostic Relevance

Pyrimidine metabolism showed the greatest statistical correlation with patient survival out of the 83 metabolic pathways examined by univariate Cox regression (Figure 3a). We looked at the prognostic significance of acetylated genes within the pyrimidine pathway because of the regulatory function that acetylation plays in cancer metabolism. Expression analysis of acetylation‐related genes revealed 30 significantly deregulated genes between tumor and adjacent normal samples (Figure 3b). These genes were cross‐referenced with 58 genes involved in pyrimidine metabolism, resulting in a correlation heatmap (Figure 3c). From this, 47 gene pairs showed correlation coefficients exceeding |0.3| and *p* values < 0.01. A subsequent differential expression test identified nine acetylated pyrimidine metabolism genes with log₂fold change > 1.5 and FDR‐adjusted *p* values < 0.05 (Figure 3D). Among them, three were overexpressed in Cluster 1, whereas two were underexpressed in Cluster 2 (Figure 3e).

Figure 3Pyrimidine metabolism and acetylation‐related gene expression. (a): Univariate Cox regression showing the significance of pyrimidine metabolism in survival, with *p* values and hazard ratios plotted. (b): Differential expression of acetylated genes in LUAD versus normal samples, identifying 30 significant genes. (c): Correlation heatmap of acetylated genes and pyrimidine metabolism genes with strong correlations (|*r*| > 0.3, *p* < 0.01). (d): Volcano plot showing nine differentially expressed acetylated pyrimidine metabolism genes with log₂ fold change>1.5 and adjusted *p* < 0.05. (e): Bar graph comparing expression levels of acetylated pyrimidine metabolism genes between clusters.

**Figure:**
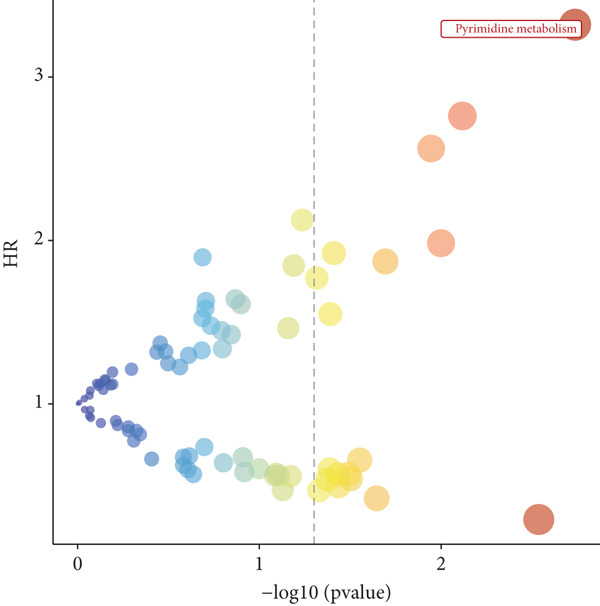
(a)

**Figure:**
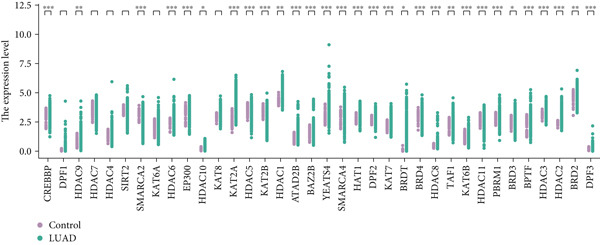
(b)

**Figure:**
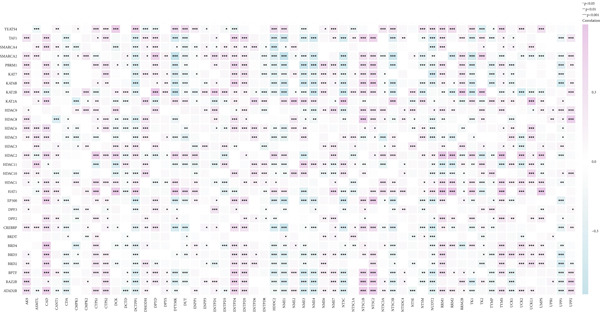
(c)

**Figure:**
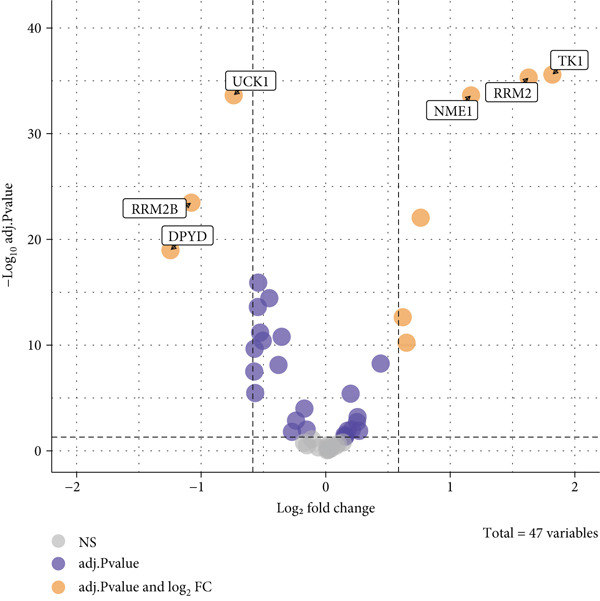
(d)

**Figure:**
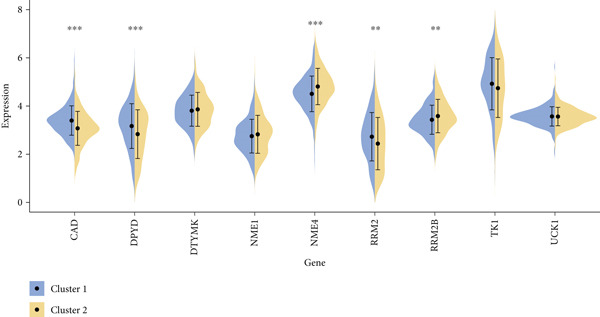
(e)

#### 2.12.4. Feature Selection of Prognostic Core Genes via Machine Learning

From the acetylated pyrimidine collection, two distinct machine learning models were used to find important prognostic genes. The model identified four top‐ranked genes using the CoxBoost method, achieving optimal performance at 68 iterations with a minimal loss value of −909.75 (Figure 4a). In parallel, a random forest survival model with an out‐of‐bag error rate of 0.4116 performed best at 23 trees (Figure 4b,c). Three overlapping core genes were identified by Venn diagram intersection analysis: TK1, RRM2B, and NME4 (Figure 4D). Functional interaction between these proteins was validated by PPI analysis based on the STRING database (Figure 4e). Differential expression plots showed NME4 and TK1 were significantly upregulated in tumor tissues, whereas RRM2B exhibited higher expression in normal samples (Figure 4f). Expression correlations demonstrated a strong inverse relationship between RRM2B and the other two genes (Figure 4g).

Figure 4Machine learning model for prognostic gene selection. (a): CoxBoost analysis showing optimal performance at 68 iterations with four key genes, achieving a partial log‐likelihood of −909.75. (b): Random forest analysis showing the optimal model with 23 trees and lowest out‐of‐bag error (0.4116). (c): Variable importance plot from random forest analysis showing key prognostic genes. (d): Venn diagram of genes identified by CoxBoost and random forest, with three overlapping core genes: NME4, RRM2B, and TK1. (e): Protein–protein interaction (PPI) network showing significant interactions between NME4, RRM2B, and TK1. (f): Box plots comparing expression of NME4 and TK1 in LUAD and control samples, and RRM2B in normal tissues. (g): Correlation heatmap showing significant negative correlations between RRM2B and the other two genes.

**Figure:**
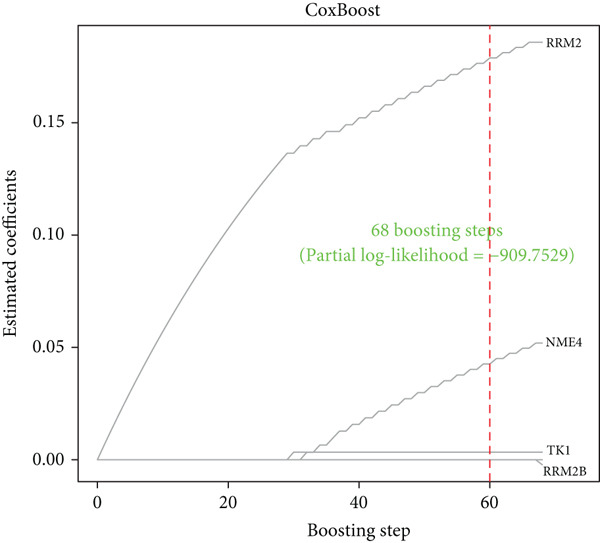
(a)

**Figure:**
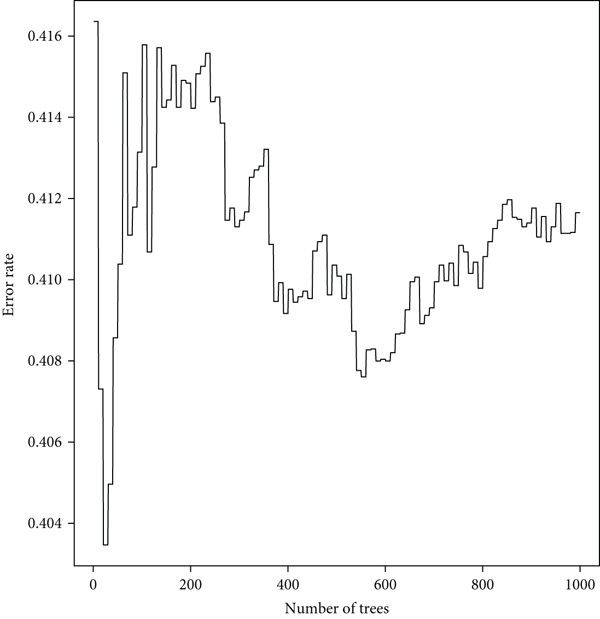
(b)

**Figure:**
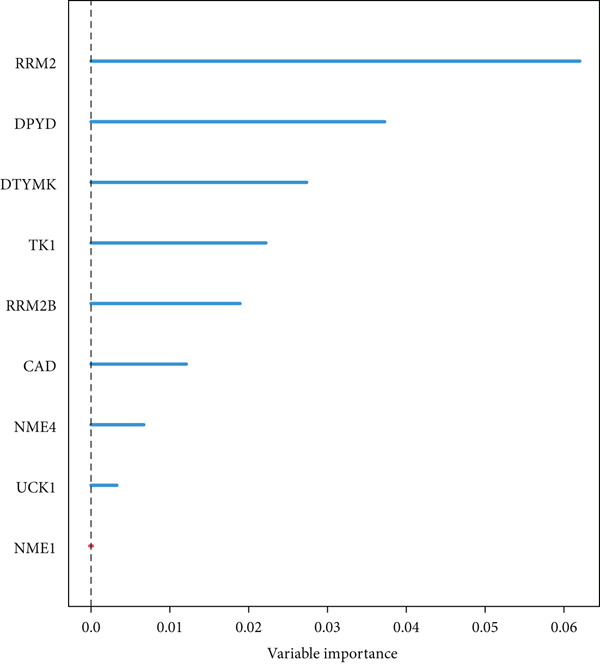
(c)

**Figure:**
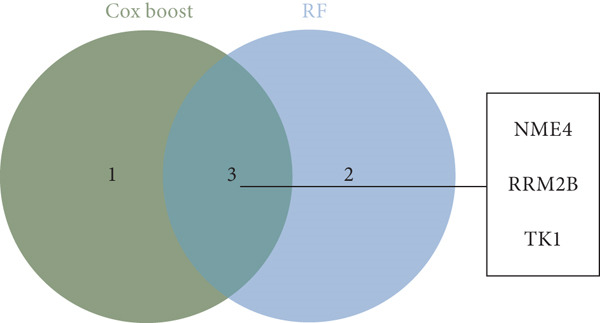
(d)

**Figure:**
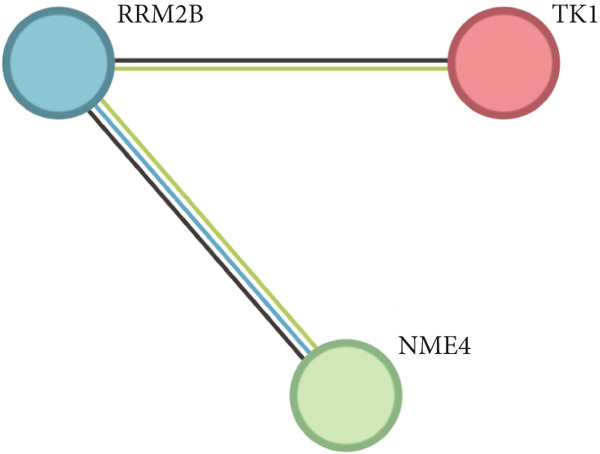
(e)

**Figure:**
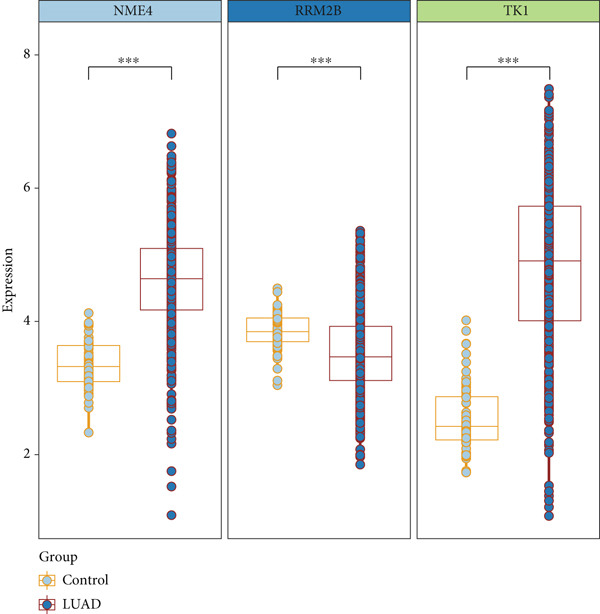
(f)

**Figure:**
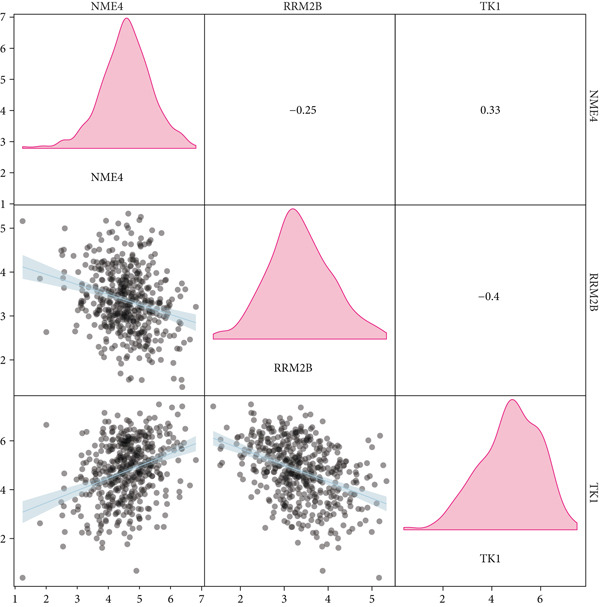
(g)

#### 2.12.5. Construction of Multivariate Prognostic Model and Risk Stratification

The three main genes were included in a multivariate Cox model that produced individual risk ratings for each patient. According to Kaplan–Meier curves, the survival rate was significantly higher for patients in the low‐risk category (Figure 5a). Strong predictive performance for overall survival at 1, 3, and 5 years was found using time‐dependent ROC analysis (Figure 5b). The gene expression patterns of the high‐ and low‐risk groups were further separated by t‐SNE analysis (Figure 5c).

Figure 5Risk model validation and prognostic performance. (a): Kaplan–Meier survival curves stratified by risk scores from multivariate Cox regression with TK1, RRM2B, and NME4 genes. Low‐risk patients show significantly better survival. (b): Time‐dependent ROC curves for 1‐, 3‐, and 5‐year survival, showing the model′s predictive accuracy. (c): t‐SNE plot clustering high‐risk and low‐risk patients based on gene expression patterns. (d–f): Validation of the risk score model using the GSE30219 dataset, showing consistent survival predictions. (g–i): Model validation in the GSE41271 dataset, confirming the robustness of the prognostic risk score.

**Figure:**
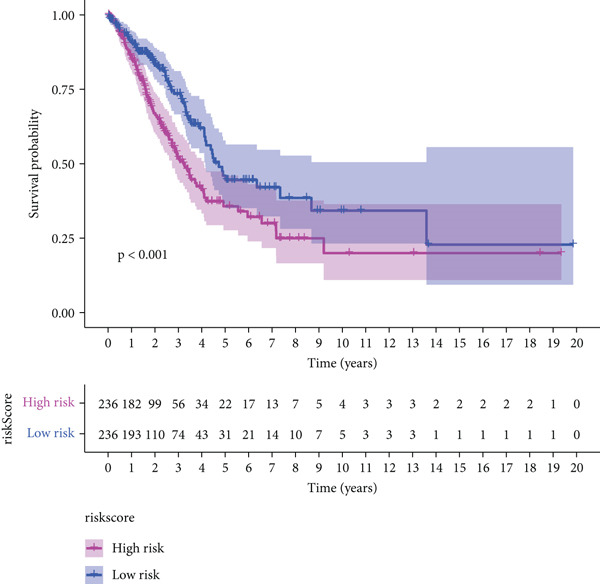
(a)

**Figure:**
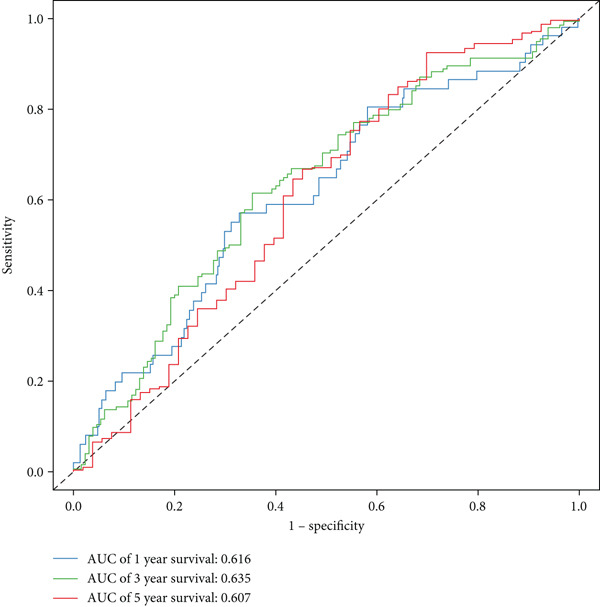
(b)

**Figure:**
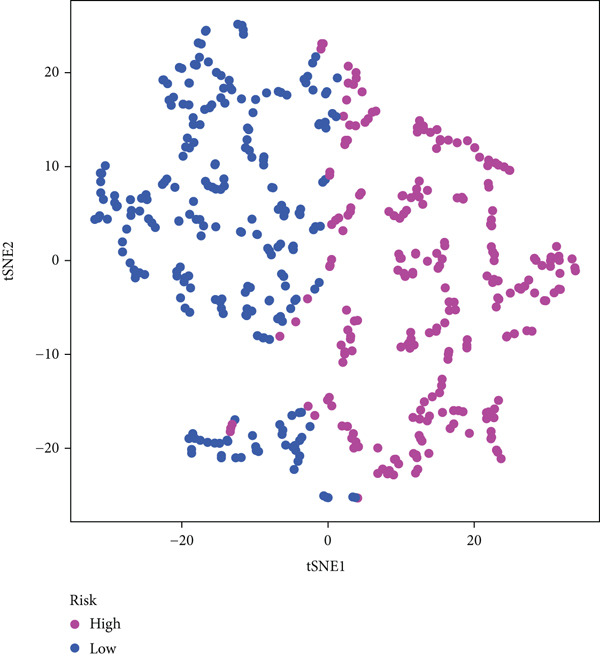
(c)

**Figure:**
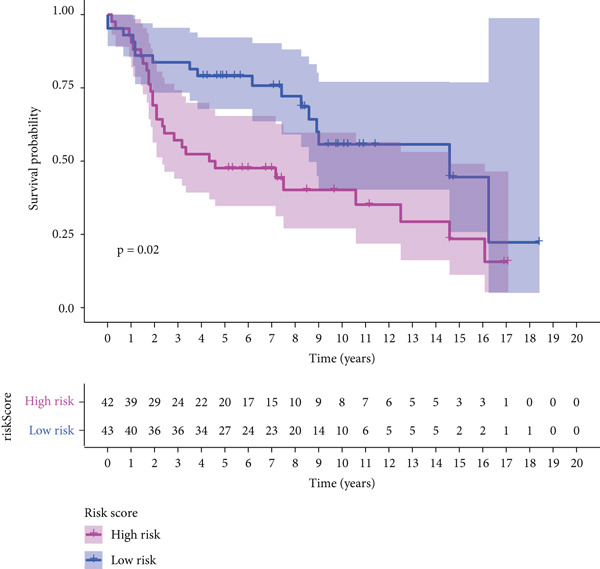
(d)

**Figure:**
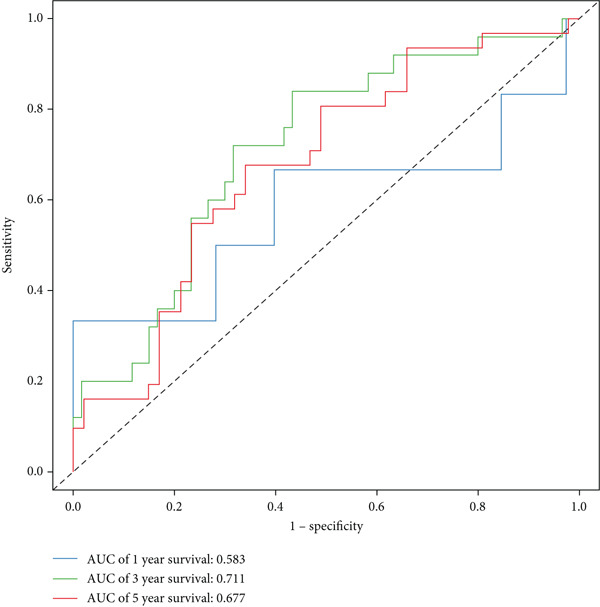
(e)

**Figure:**
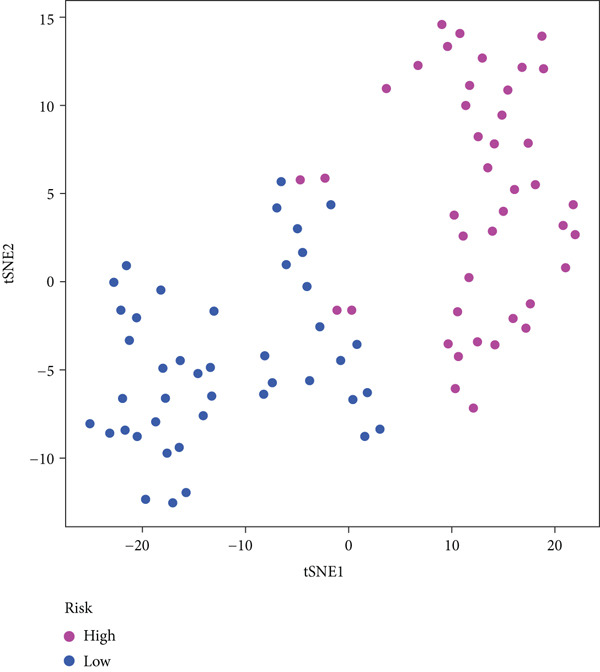
(f)

**Figure:**
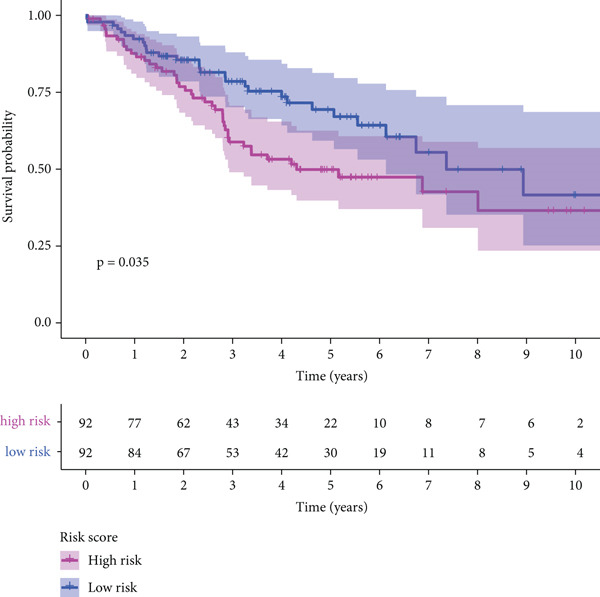
(g)

**Figure:**
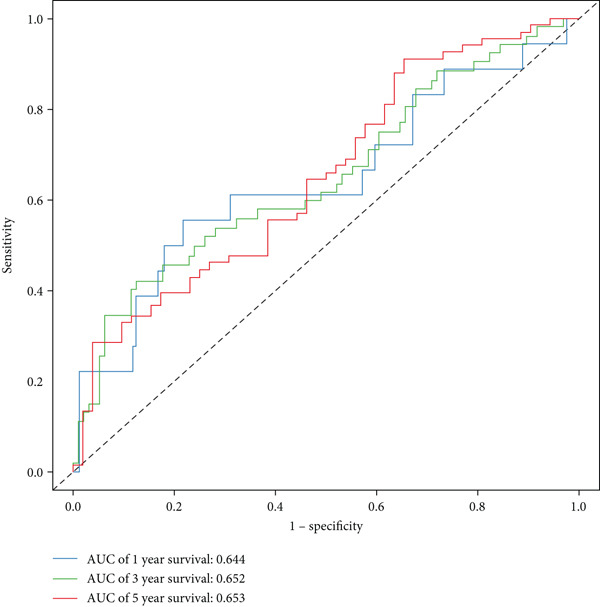
(h)

**Figure:**
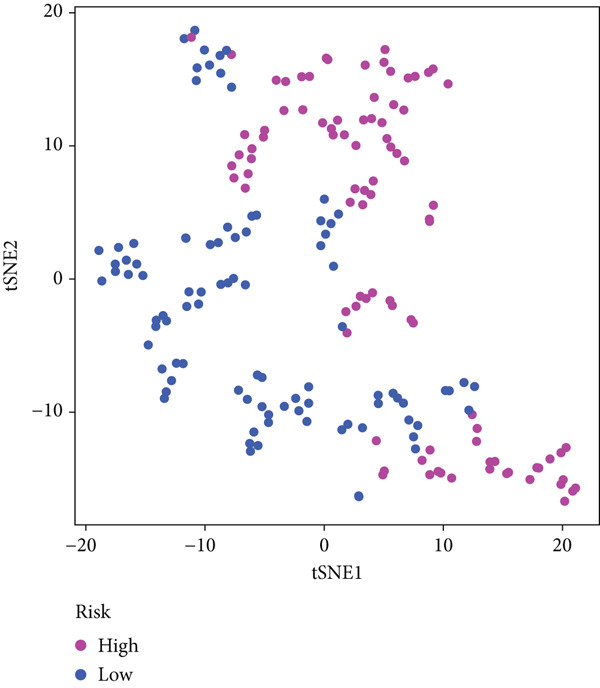
(i)

#### 2.12.6. External Validation Using Independent GEO Cohorts

To verify the repeatability of the model, GSE30219 and GSE41271 datasets were used. The three‐gene model′s generalizability was reinforced by stratified analysis of both cohorts, which verified consistent survival trends, ROC curves and t‐SNE clustering patterns as shown in the training dataset (Figures 5d, 5e, 5f, 5g, 5h, and 5i).

#### 2.12.7. Immune Microenvironment Stratification Between Risk Groups

To examine the tumor microenvironment between the high‐ and low‐risk groups, immune profile analysis was employed. The CIBERSORTx analysis revealed that the low‐risk group had higher levels of resting memory CD4^+^ T cells, plasma cells, and naïve B cells infiltrated. On the other hand, high‐risk tumors had higher concentrations of M1/M2 macrophages, activated CD4^+^ memory T cells, and CD8^+^ T cells (Figure 6a,b). Using the IOBR software, 220 curated signatures were subjected to ssGSEA‐based immune signature scoring. Signatures that were abundant in the high‐risk group included hypoxia, Th2 cell activity, and MDSCs, all of which were linked to increased hazard ratios according to univariate Cox regression (Figure 6c,d). TK1 and NME4 were shown to have favorable correlations with immunosuppressive or tumor‐promoting immunological characteristics, according to correlation matrices (Figure 6e).

Figure 6Immune profiling in high‐ and low‐risk groups. (a,b): CIBERSORT analysis showing immune cell composition differences between low‐ and high‐risk groups, with higher CD8^+^ T cells and macrophages in high risk and more B cells and plasma cells in low risk. (c,d): ssGSEA analysis of immune signatures in high‐risk groups, highlighting pathways like hypoxia and Th2 cells associated with worse prognosis. (e): Correlation heatmap showing associations between core genes (TK1, RRM2B, and NME4) and immune markers with HR > 1.

**Figure:**
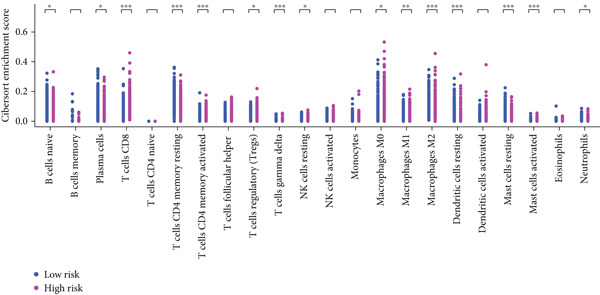
(a)

**Figure:**
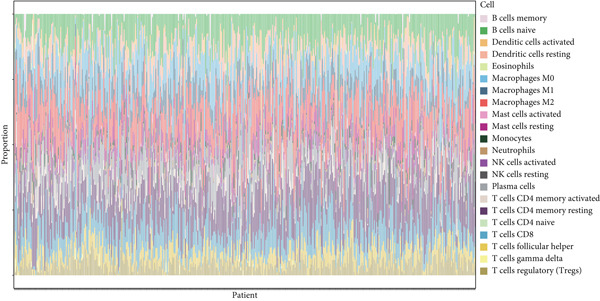
(b)

**Figure:**
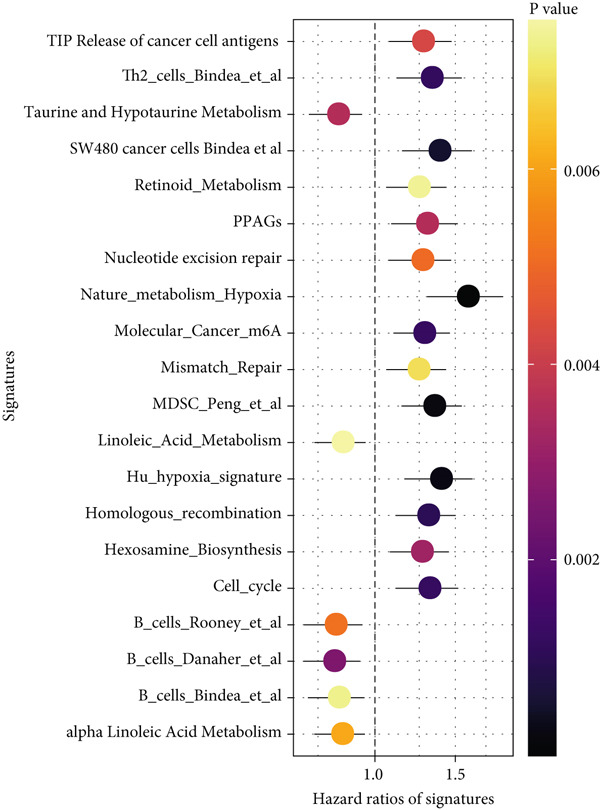
(c)

**Figure:**
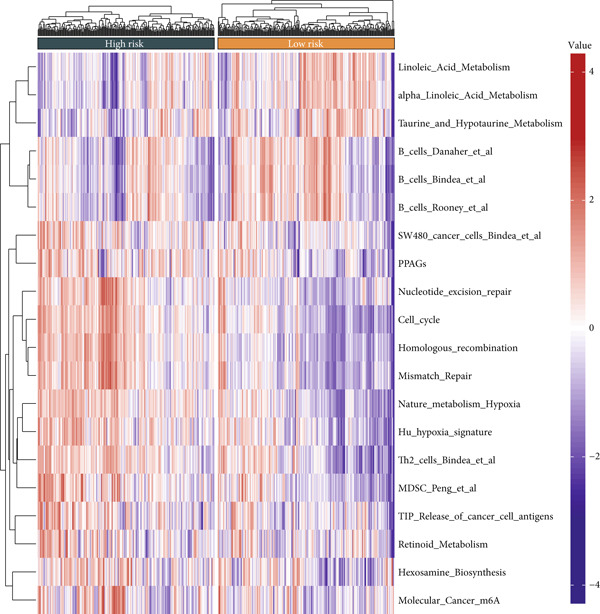
(d)

**Figure:**
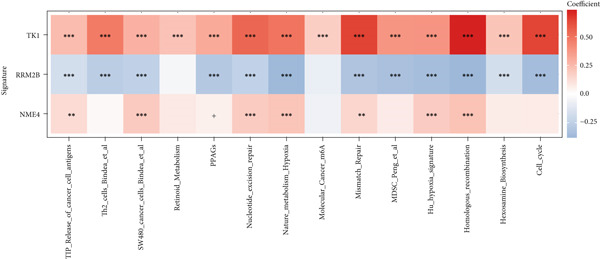
(e)

#### 2.12.8. Immunotherapy Response Prediction Using ARPG Scoring

To assess potential response to immunotherapy, an ARPG score was computed from the expression values of NME4, RRM2B, and TK1. Analysis of the IMvigor210 dataset showed that individuals with lower ARPG scores had superior response rates 3 months after treatment initiation (Figure 7a,b). Stratification based on immune response classification demonstrated that ARPG scores of patients with partial response (PR) or complete response (CR) were considerably lower than those with progressing illness (PD) (Figure 7c). GSE78220, GSE91061, and GSE135222 were among the additional immunotherapy groups that verified the constant correlation between lower ARPG scores and improved overall survival and responsiveness to treatment (Figures 7d, 7e, and 7f). Furthermore, the low‐risk group had a much greater expression of immune checkpoint molecules, suggesting that their tumor profile may be more immune responsive (Figure 7g).

Figure 7Immunotherapy response and ARPG scores. (a,b): Kaplan–Meier survival curves at 3 months posttreatment comparing overall survival in the IMvigor210 cohort based on high and low ARPG scores. (c): Bar graph showing ARPG scores by immune response category (CR, PR, and PD), with lower scores correlating with better responses. (d,e): Survival analysis in additional immunotherapy cohorts (GSE78220 and GSE91061), confirming the association between low ARPG scores and improved survival. (f): Immune checkpoint expression analysis, showing higher checkpoint expression in the low‐risk group, indicating better immunotherapy response. (g): Expression of immune checkpoint molecules (e.g., PD‐1 and CTLA‐4) in ARPG risk groups.

**Figure:**
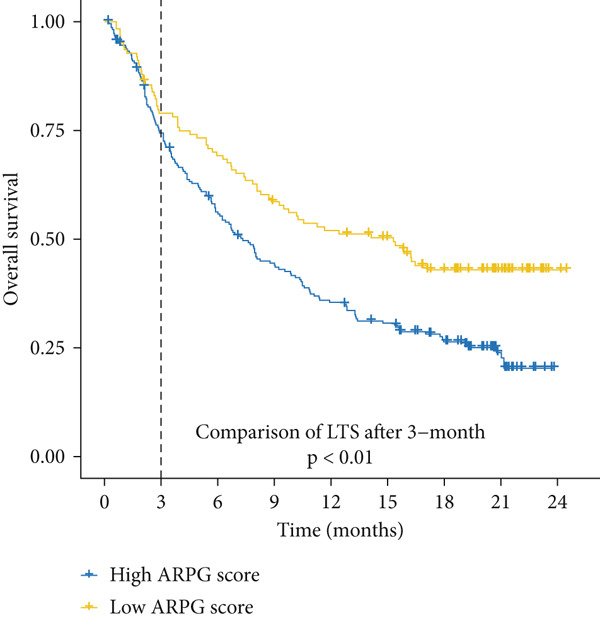
(a)

**Figure:**
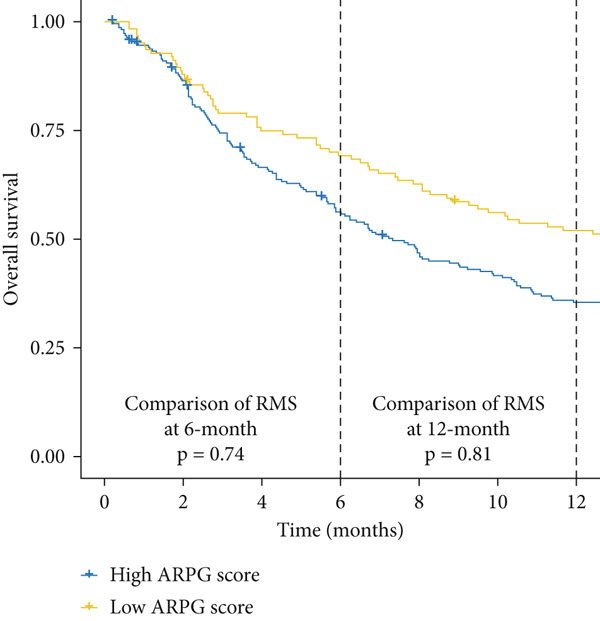
(b)

**Figure:**
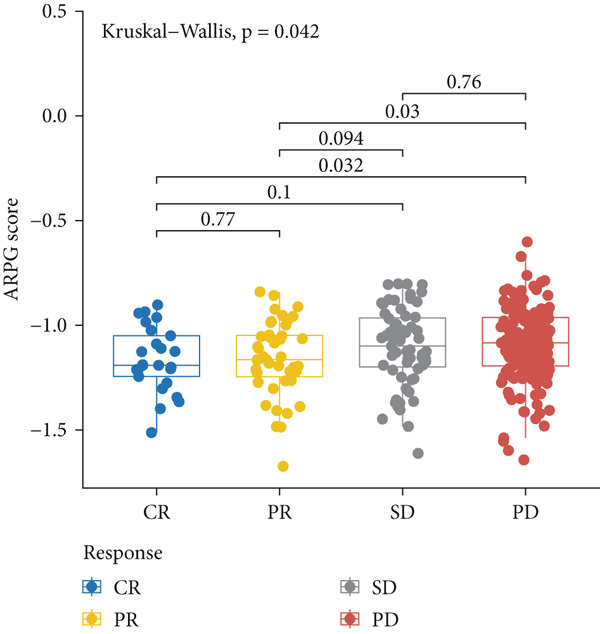
(c)

**Figure:**
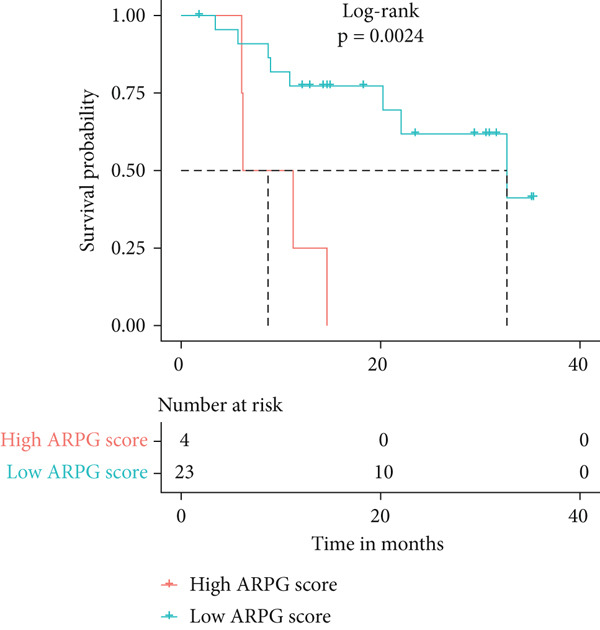
(d)

**Figure:**
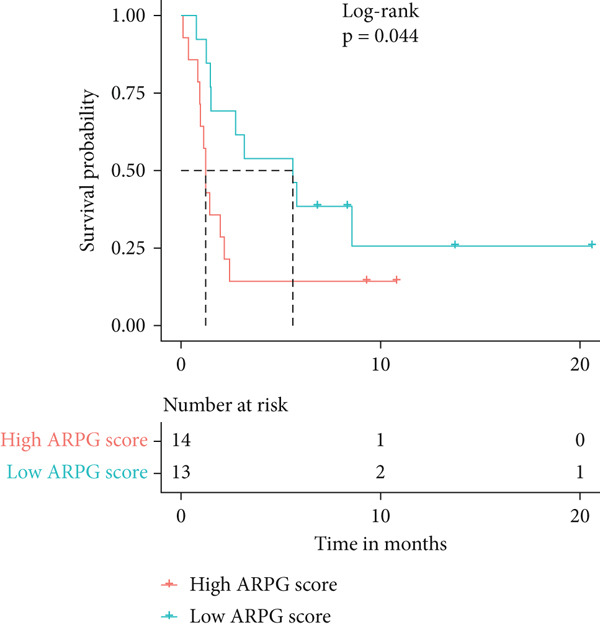
(e)

**Figure:**
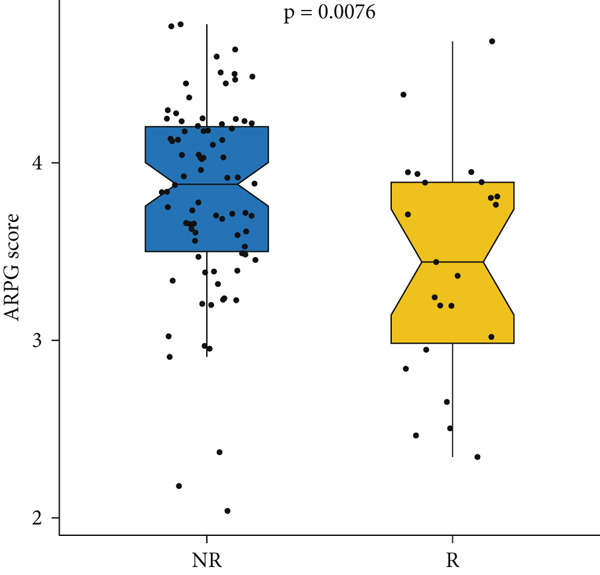
(f)

**Figure:**
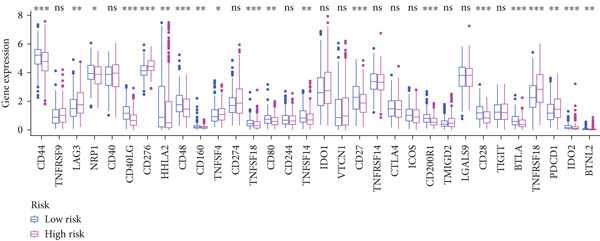
(g)

#### 2.12.9. Chemosensitivity Estimation Based on Risk Stratification

Using pharmacogenomic analysis and the pRRophetic algorithm, medications with varying levels of efficacy across risk categories were found. Four substantial positive correlations between the risk score and the Top 9 candidate drugs indicated greater effectiveness in the low‐risk category. The remaining five showed negative correlations, implying greater suitability for high‐risk patients (Figures 8a, 8b, 8c, 8d, 8e, 8f, 8g, 8h, and 8i).

Figure 8Drug sensitivity analysis based on risk scores. (a–i): Drug sensitivity plots for Top 9 drugs using pRRophetic analysis, correlating drug sensitivity with risk scores.

**Figure:**
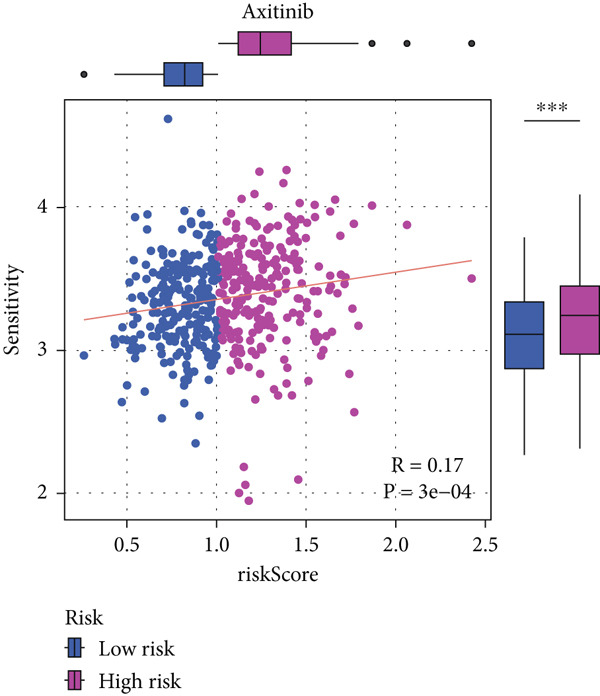
(a)

**Figure:**
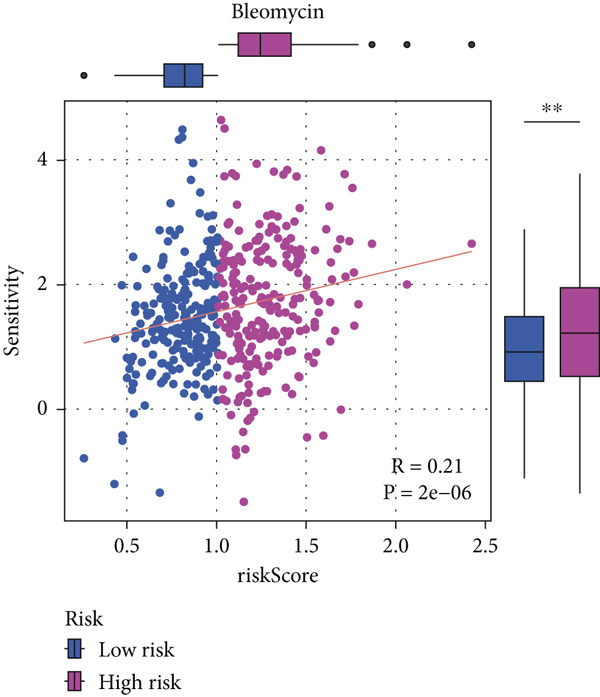
(b)

**Figure:**
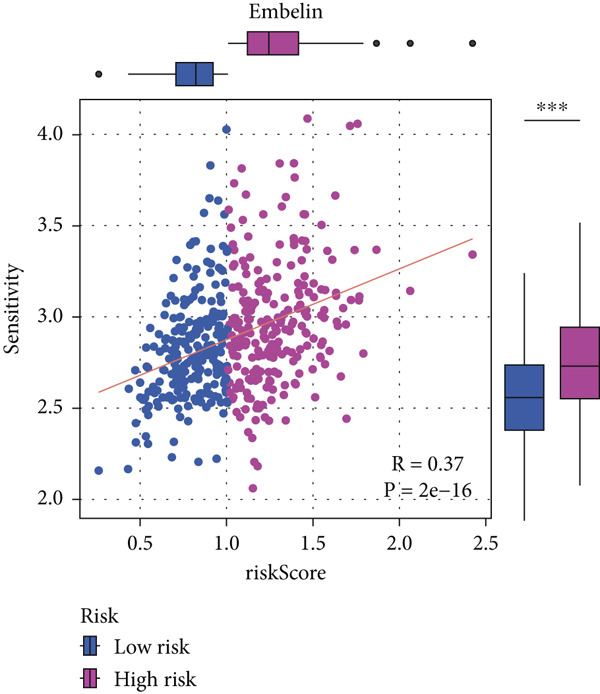
(c)

**Figure:**
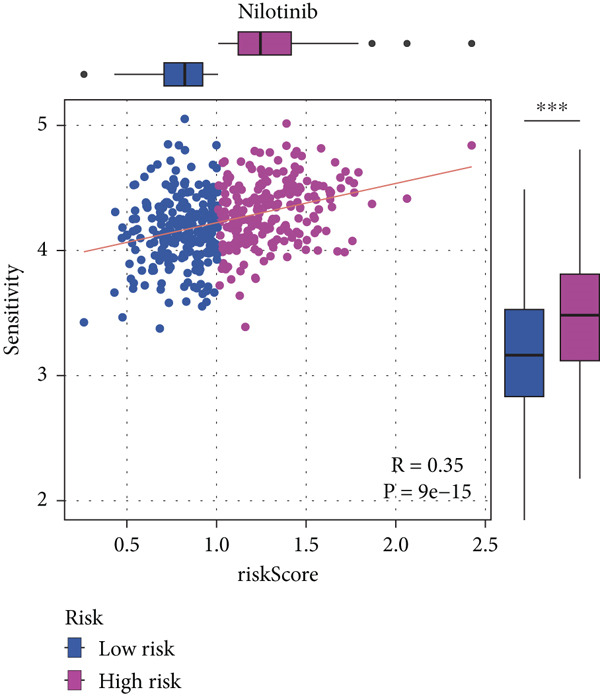
(d)

**Figure:**
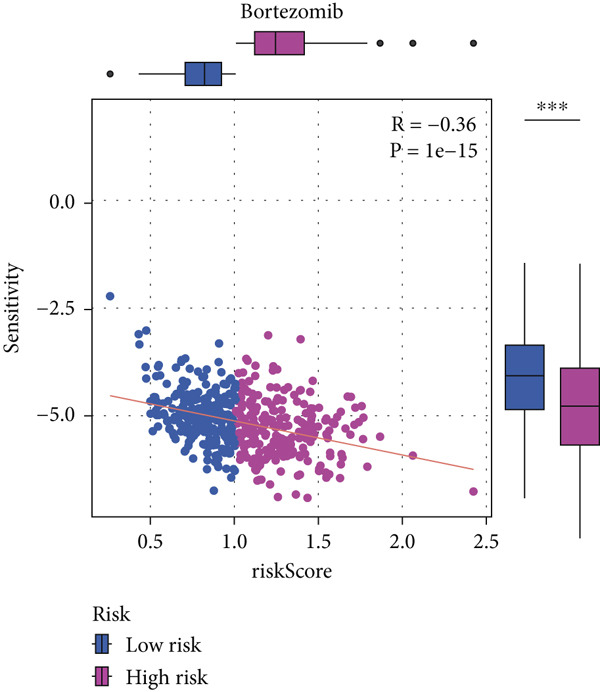
(e)

**Figure:**
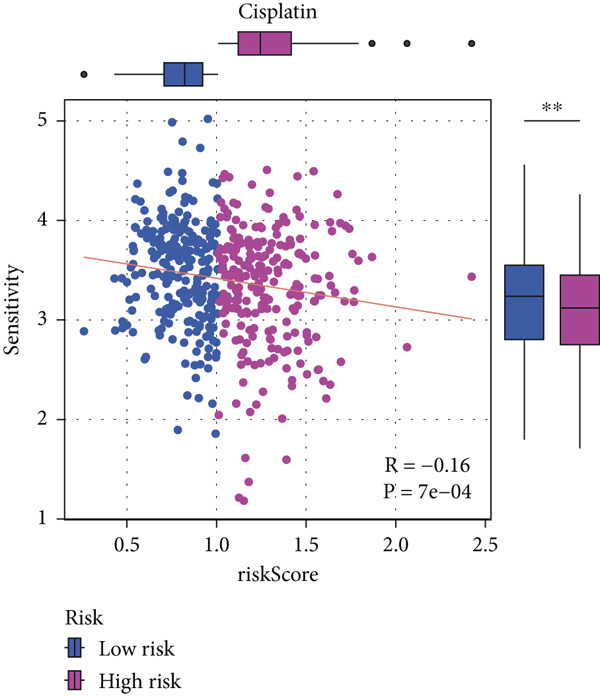
(f)

**Figure:**
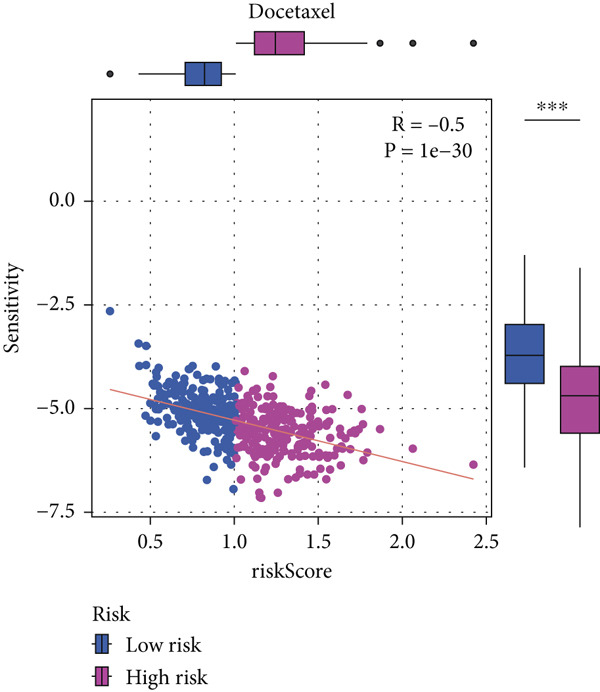
(g)

**Figure:**
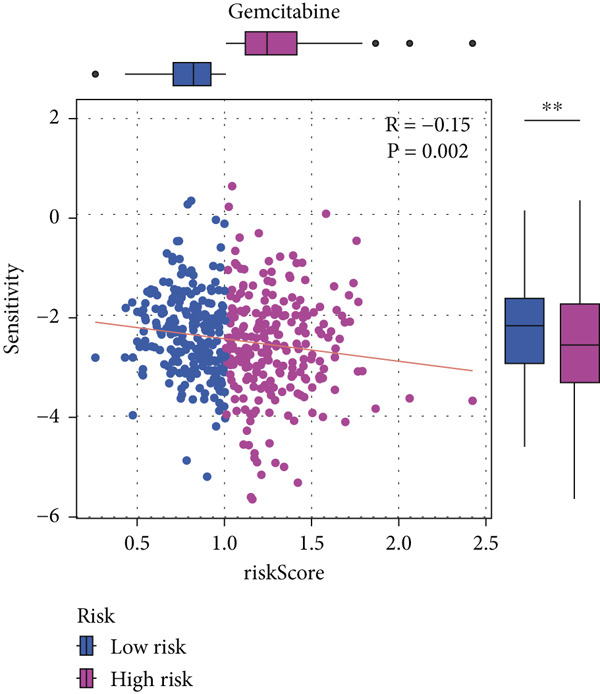
(h)

**Figure:**
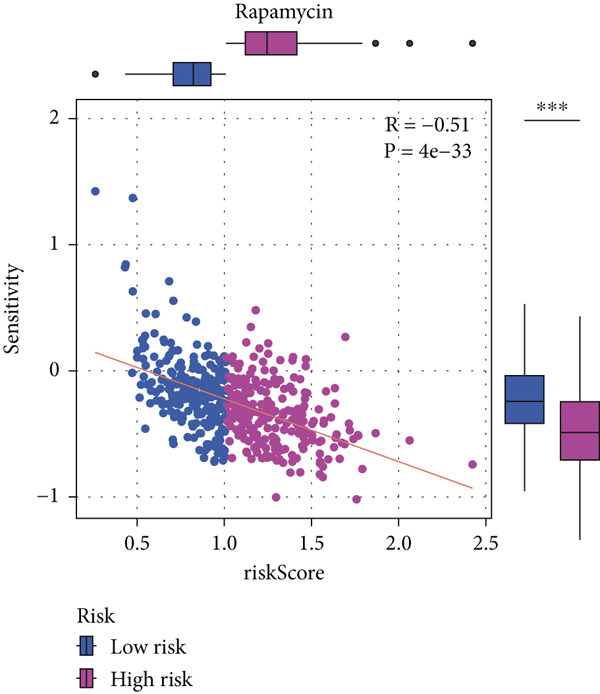
(i)

#### 2.12.10. Clinical Integration and Nomogram Development

Clinical variables including age, sex, TNM stage, and calculated risk scores were incorporated into a univariate Cox regression model. Both TNM staging and risk score emerged as independent predictors of overall survival (*p* < 0.05) (Figure 9a). To assess survival probability at 1, 3, and 5 years, a prognostic nomogram was created by combining clinical and molecular data (Figure 9b). Strong model performance was shown by calibration plots and ROC curves in the TCGA cohort (Figure 9c,d), and the GSE30219 cohort was used to confirm similar accuracy (Figure 9e,f).

Figure 9Clinical outcome modeling and prognostic nomogram. (a): Univariate Cox regression analysis of clinical parameters and risk score, with *p* values and hazard ratios indicating significant survival associations. (b): Nomogram for predicting 1‐, 3‐, and 5‐year survival probabilities based on clinical parameters and risk score. (c–f): Calibration and ROC curves assessing nomogram accuracy in the training and validation sets (GSE30219), showing a strong predictive performance.

**Figure:**
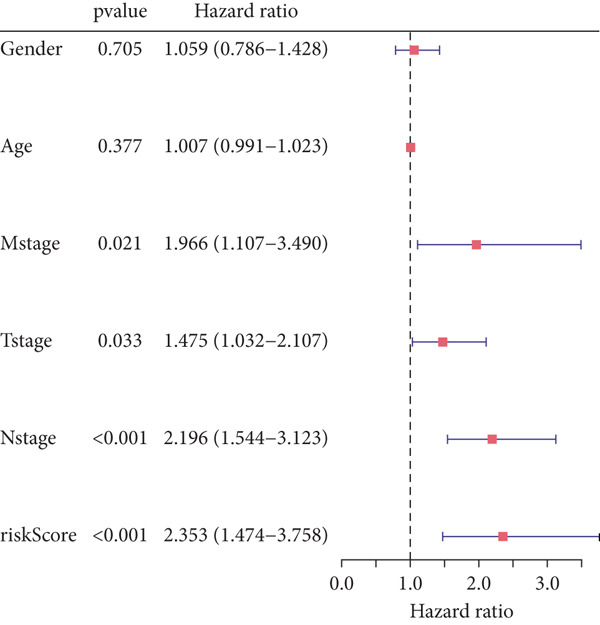
(a)

**Figure:**
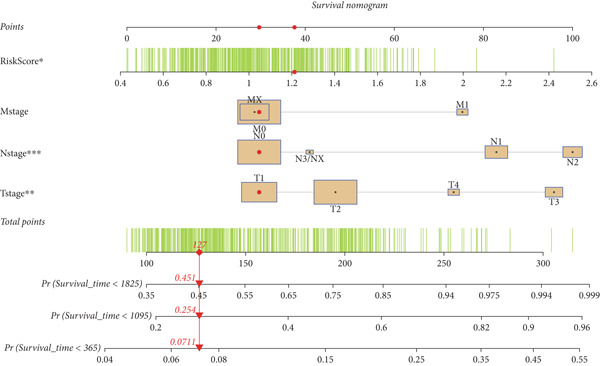
(b)

**Figure:**
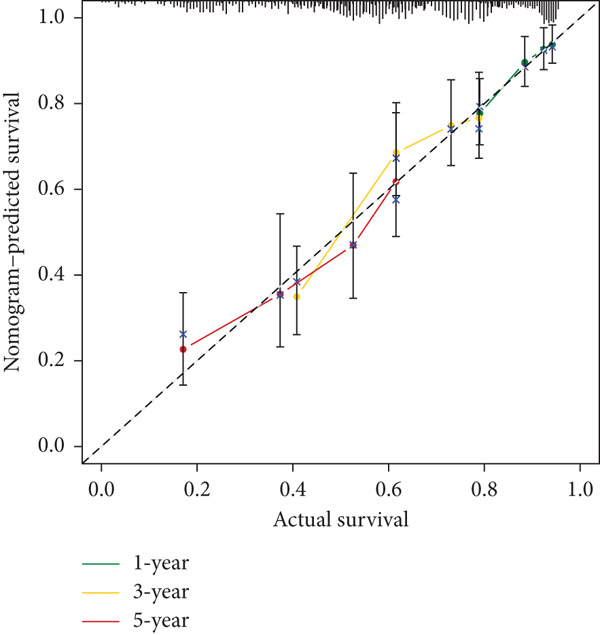
(c)

**Figure:**
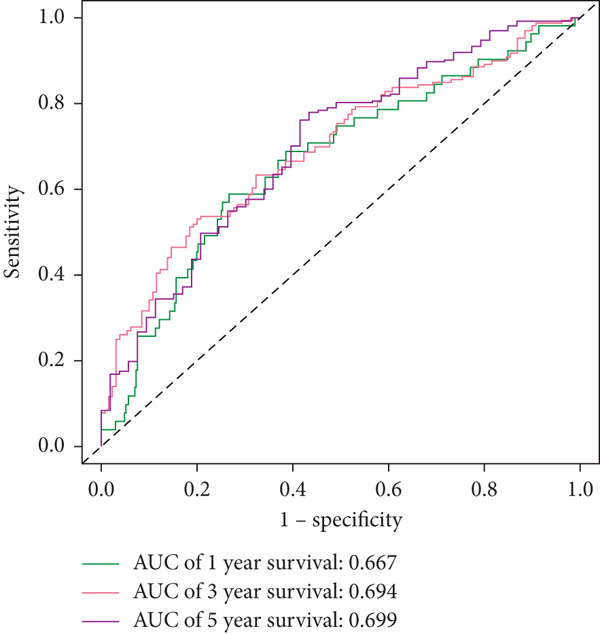
(d)

**Figure:**
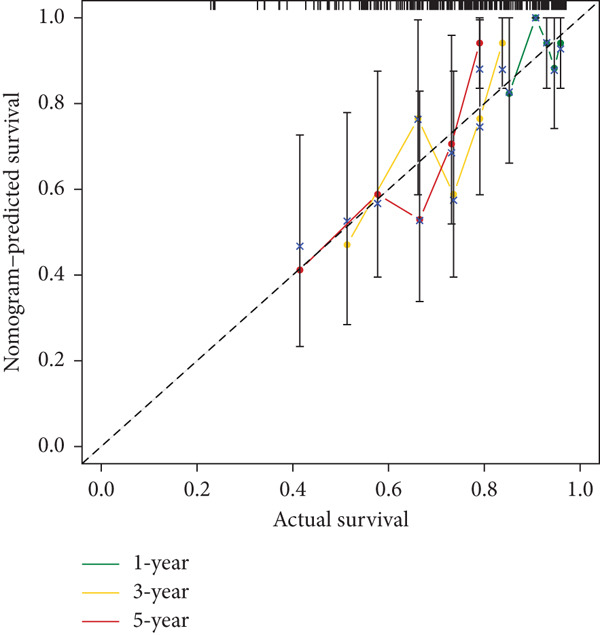
(e)

**Figure:**
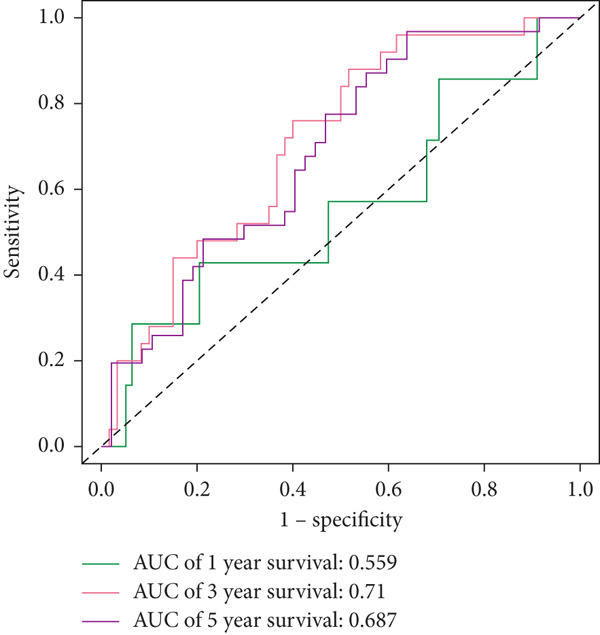
(f)

#### 2.12.11. Functional Role of TK1 in Lung Adenocarcinoma Cells

The carcinogenic role of TK1 was confirmed using in vitro silencing studies. RT‐qPCR analysis confirmed robust TK1 knockdown efficacy (*p* < 0.001), Figure 10a). CCK‐8 assays revealed a significant drop in cell viability after TK1 inhibition (*p* < 0.001, Figure 10b). EdU incorporation assays showed that the proliferation of TK1‐silenced cells was considerably lower than that of controls (*p* < 0.001, Figure 10c). Significant decreases in invasive capacity were seen after a knockdown in invasion studies employing Matrigel‐coated Transwell inserts (*p* < 0.001, Figure 10D). Furthermore, the TK1 knockdown group exhibited a delayed closure in wound healing experiments, suggesting defective migration (*p* < 0.001, Figure 10e). Together, our findings show that TK1 is essential for enhancing lung adenocarcinoma cell motility, invasion, and proliferation.

Figure 10In vitro validation of TK1 function in LUAD cells. (a): RT‐qPCR confirming efficient knockdown of TK1 expression in LUAD cells. (b): CCK‐8 assay showing significantly reduced cell viability in the TK1 knockdown group compared with controls. (c): EdU incorporation assay demonstrating decreased proliferation in TK1‐silenced cells. (d): Transwell invasion assay showing reduced invasion of TK1‐knockdown cells compared with controls. (e): Wound healing assay revealing delayed migration of TK1‐silenced cells, indicating impaired cell motility.

**Figure:**
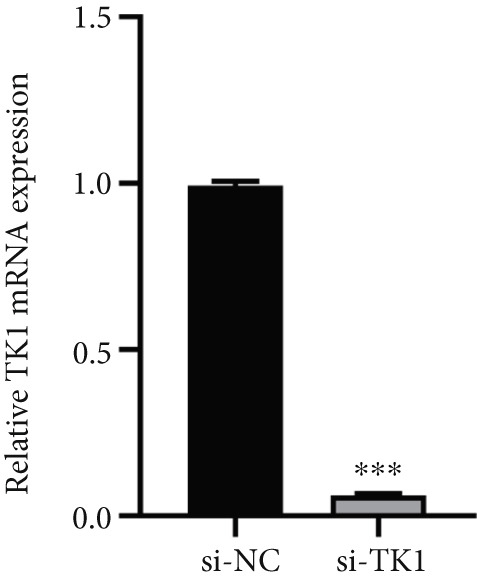
(a)

**Figure:**
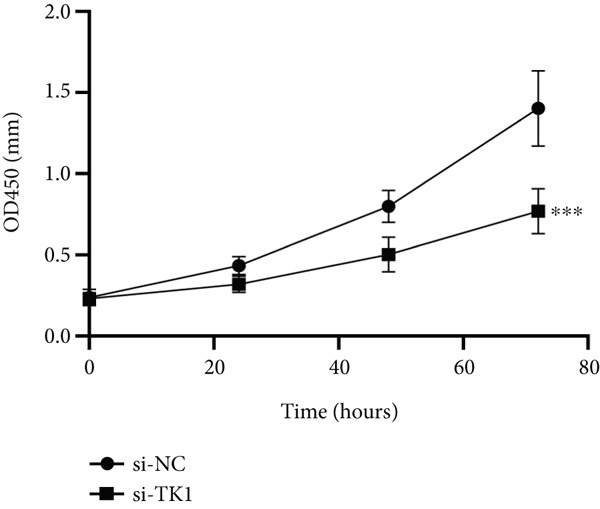
(b)

**Figure:**
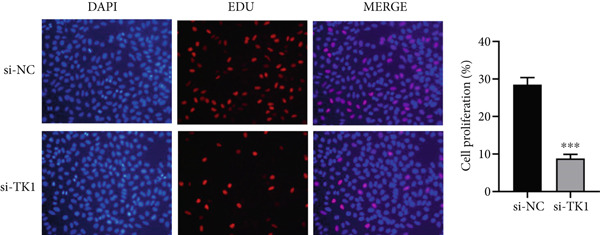
(c)

**Figure:**
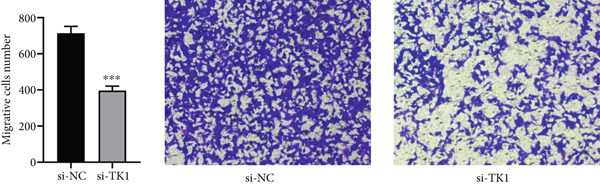
(d)

**Figure:**
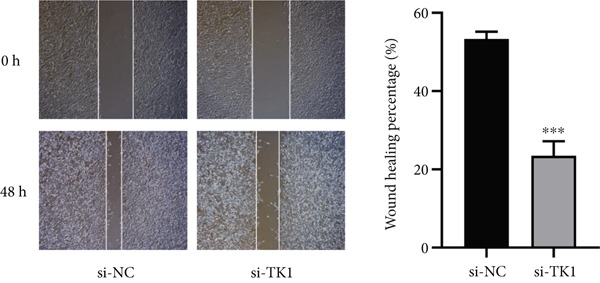
(e)

## 3. Discussion

The current study provides a detailed exploration of the role of acetylated pyrimidine metabolism genes in LUAD and highlights their potential as biomarkers for prognosis and therapeutic decision‐making. In this study, we identified TK1, NME4, and RRM2B as critical genes involved in pyrimidine metabolism that are associated with overall survival in LUAD. These findings emphasize the importance of metabolic regulation, particularly posttranslational modifications like acetylation, in influencing tumor progression, immune microenvironment modulation, and treatment responses.

Our results position TK1 as a central driver of LUAD progression. Consistent with previous studies, TK1 has been shown to promote cell proliferation, migration, and invasion across various cancers, including liver and pancreatic cancer [30, 31]. In our in vitro experiments, silencing of TK1 significantly impaired LUAD cell proliferation and migration, suggesting that TK1′s enzymatic activity and its role in maintaining dTMP synthesis are central to its oncogenic properties. Additionally, TK1 has been identified as a key gene associated with Treg cell infiltration and triple‐negative breast cancer (TNBC) progression [32], highlighting its involvement in immune modulation. Furthermore, pan‐cancer analyses have shown that TK1 facilitates tumor progression by promoting Th2 cell polarization, which contributes to an immunosuppressive microenvironment [33]. These immune‐related functions suggest that TK1 could serve as a promising therapeutic target, potentially in combination with immune checkpoint inhibitors to enhance treatment efficacy.

Moreover, TK1 appears to contribute to metabolic reprogramming through interaction with PRMT1, as reported in hepatocellular carcinoma, where it promotes glycolysis and tumor aggressiveness [30]. The interaction between TK1 and PRMT1 in LUAD warrants further investigation to explore potential therapeutic avenues targeting this axis.

Interestingly, our study also highlights acetylation as a significant modulator of TK1 expression. Previous studies have shown that TK1 expression is regulated by mechanisms such as m6A modification [34]. Our findings extend this knowledge by demonstrating that acetylation may represent an additional layer of regulation, influencing TK1 expression and activity in LUAD. This adds complexity to the posttranslational regulatory network underlying TK1′s oncogenic role and suggests that targeting acetylation could offer a novel approach to modulate TK1′s activity.

NME4, another gene identified in this study, has been implicated in mitochondrial function and tumor metastasis. Consistent with the literature, NME4 interacts with the dynamin‐related protein OPA1, which plays a crucial role in mitochondrial fusion [35]. Studies suggest that mitochondrial fusion inhibits metastasis, whereas mitochondrial fission promotes it. Our results support this concept, as NME4 overexpression in LUAD was associated with enhanced mitochondrial fusion and reduced metastatic potential. Additionally, NME4 modulated the immune microenvironment through the NF*κ*B2–CCL5 axis, which restricts CD8^+^ T cell tumor infiltration in squamous cell carcinoma [36].

Furthermore, recent studies using multiplex immunofluorescence have demonstrated spatial co‐localization and exclusion patterns between NME4 and immune cells, such as CD3^+^ T cells and CD20^+^ B cells [37]. Functional assays, both in vitro and in vivo, showed that NME4 regulates LUAD cell proliferation and invasion. Notably, NME4 inhibition led to increased CD8^+^ T cell abundance and activation, enhancing the antitumor immune response and improving the efficacy of anti–PD‐1 therapy. These findings position NME4 as a potential target for combination therapies with immune checkpoint inhibitors, offering a promising strategy for enhancing immune‐mediated tumor elimination.

In addition, NME4 regulates PD‐L1 expression through the STAT3 signaling pathway, which is associated with immune evasion in aggressive cancers [38]. Our study supports this, as elevated NME4 expression in LUAD correlates with immune suppression. Targeting NME4 to restore immune surveillance in LUAD could provide a novel strategy to potentiate the effects of immunotherapy.

RRM2B, a key enzyme in ribonucleotide reductase activity, was also identified as a prognostic marker in LUAD. The importance of RRM2B in maintaining DNA integrity and responding to replication stress is well‐established [39]. In our study, RRM2B exhibited differential expression patterns, with higher expression in normal tissues compared with LUAD samples, which is consistent with its role in DNA repair. In normal tissues, RRM2B likely supports genomic stability, whereas its reduced expression in LUAD may contribute to tumorigenesis. Recent studies have suggested that RRM2B interacts with p53 to prevent replication stress and maintain dNTP pools [40], raising important questions about how acetylation affects RRM2B′s role in DNA repair and genomic stability.

A major finding in our study was the differential immune landscape between the low‐risk and high‐risk groups based on TK1, NME4, and RRM2B expression. Low‐risk patients exhibited higher infiltration of favorable immune subsets, such as plasma cells, naïve B cells, and resting memory CD4^+^ T cells, whereas high‐risk patients exhibited a tumor‐promoting immune signature, enriched for M1/M2 macrophages and CD8^+^ T cells. These findings suggest that metabolic alterations in TK1, NME4, and RRM2B not only contribute to tumor progression but also shape the immune microenvironment, influencing response to immunotherapy. This aligns with emerging research on metabolism‐immune crosstalk in cancer, where metabolic changes impact immune cell infiltration and tumor immune evasion [41].

Our study further confirmed the potential of acetylation profiling as a predictor of immunotherapy response, particularly in cohorts such as IMvigor210 and GSE91061, where lower ARPG scores were correlated with improved outcomes. These findings are in line with recent studies suggesting that metabolic and acetylation profiles can serve as predictive markers for immunotherapy efficacy [42, 43]. Given the increasing use of immune checkpoint inhibitors in LUAD, incorporating acetylation‐based metabolic profiling into clinical practice could significantly improve treatment stratification and outcomes.

## 4. Limitations and Future Directions

Although our findings underscore the prognostic value of acetylated pyrimidine metabolism genes in LUAD, several questions remain. First, the role of acetylation in modulating TK1, NME4, and RRM2B warrants further mechanistic exploration, particularly in relation to other posttranslational modifications like phosphorylation. Additionally, factors such as EGFR mutation status, smoking history, and PD‐L1 expression, which influence LUAD prognosis and treatment response, were not fully addressed in this study. Future research should incorporate these variables to assess the independent prognostic value of our biomarkers. Finally, the lack of in vivo validation limits the translational potential of our findings. In vivo models or patient‐derived xenografts will be critical for confirming the functional relevance of these metabolic genes in LUAD progression and therapeutic response.

## 5. Conclusion

In conclusion, our study offers novel insights into the prognostic and therapeutic potential of acetylated pyrimidine metabolism genes in LUAD. By integrating multiomics data, we identified TK1, NME4, and RRM2B as key biomarkers that influence tumor progression, immune modulation, and treatment response. These findings, particularly regarding TK1 and NME4′s roles in immune modulation and their potential as therapeutic targets, pave the way for personalized treatment strategies incorporating acetylation profiling and metabolic reprogramming. Such strategies could significantly improve patient outcomes in LUAD, particularly with the incorporation of immune checkpoint inhibitors.

## Disclosure

All authors reviewed and approved the final manuscript.

## Conflicts of Interest

The authors declare no conflicts of interest.

## Author Contributions

K.J. and S.Z. contributed equally to data analysis and manuscript writing. Y.H. and L.L. supervised the study, helped with data interpretation, and revised the manuscript. Y.H. also conducted the experiments. K.J. and S.Z. contributed to the work equally and should be regarded as co‐first authors.

## Funding

This study was supported by Sichuan Science and Technology Program (No.2023YFS0160).

## Data Availability

All datasets utilized or generated during the study are fully available within this article.
